# Application of the Theory of Planned Behavior in Environmental Science: A Comprehensive Bibliometric Analysis

**DOI:** 10.3390/ijerph16152788

**Published:** 2019-08-04

**Authors:** Hongyun Si, Jian-gang Shi, Daizhong Tang, Shiping Wen, Wei Miao, Kaifeng Duan

**Affiliations:** School of Economics and Management, Tongji University, Shanghai 200092, China

**Keywords:** theory of planned behavior, TPB, environmental behavior, intention, bibliometric analysis

## Abstract

Since the theory of planned behavior (TPB) was proposed by Ajzen in 1985, it has attracted extensive interest and been widely applied worldwide. Although an increasing number of studies have employed the TPB in the domain of environmental science, there have been no attempts to retrospectively analyze existing articles. The current study aimed to holistically understand the application status of the TPB in environmental science from a knowledge domain visualization perspective. A total of 531 journal articles were obtained through the Scopus database to perform a bibliometric analysis and content analysis. The results showed that waste management, green consumption, climate and environment, saving and conservation, and sustainable transportation are the primary research topics; the United States (U.S.), Mainland China, the United Kingdom (UK), and Malaysia are the most productive countries/regions. Moreover, the cross-disciplinary situations, main source journals, and key articles were revealed. Furthermore, the extended factors, integrated theories, major methods, specific groups, and control variables of environmental science research using the TPB were elaborated and integrated into a comprehensive application framework. Constructive criticisms were ultimately discussed. The findings contribute in several ways to help relevant researchers learn about the application of TPB to environmental science and provide new insights and holistic references for further research on environment-related behavior.

## 1. Introduction

According to the latest report by the Intergovernmental Panel on Climate Change [1], human behavior and activities are the main causes of global warming, which has been widely recognized as a severe challenge for the survival of humans [2,3]. To address global environmental challenges, understanding how humans determine environmentally relevant behaviors is vital [4]. Over the last decade, there has been increased interest in applying the theory of planned behavior (TPB) to environmental science research worldwide, since it can provide valuable implications not only for predicting and managing individual behavior, but also for increasing social and environmental sustainability [5,6,7].

**Figure 1:**
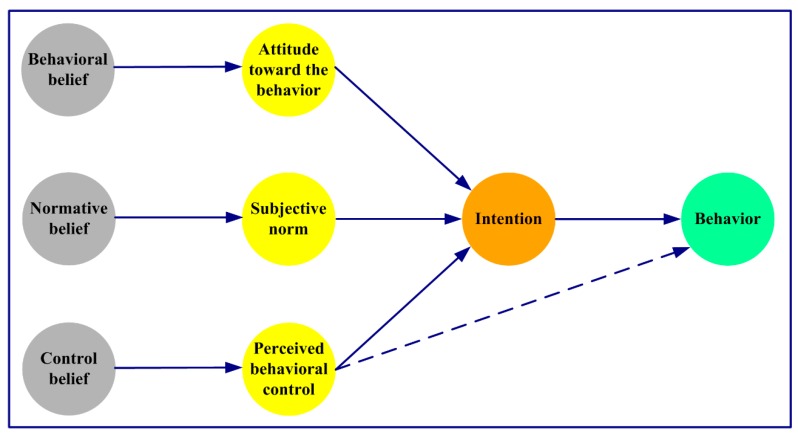
Theory of planned behavior.

The predecessor of the TPB is the theory of reasoned action (TRA) [8], which posits that the attitude and subjective norms are the determinants of intention, and that intention directly affects behavior to some extent. The TRA assumes that individual behavior is controlled by volition, while the skills, resources, or opportunities needed to perform a particular behavior are not considered. Ajzen incorporated the variable “perceived behavioral control” and corresponding “control belief” into the TRA, and then proposed the TPB (see Figure 1) [9]. In the TPB, individual intention mainly depends on three determinants: attitude, subjective norms, and perceived behavioral control. These determinants are all based on corresponding underlying belief structures: behavioral beliefs, normative beliefs, and control beliefs [10]. In addition, individual behavior is described as a function of intention and perceived behavioral control [11]. After more than 20 years of application and extension, the TPB has become one of the most important theories in social psychology that is used to elaborate the determinants of individual decision making [12]. Especially in the field of environmental science, the TPB is increasingly being advocated as a key theory for predicting and promoting a variety of pro-environmental behaviors [4,7]. With the rapid growth of the world’s population, investigating human behavior and psychology is increasingly important for environmental sustainability. Thus, it is necessary to look back at the application and development status of the TPB in the field of environmental science in order to provide a holistic and valuable information basis for follow-up researchers.

Previous scholars have investigated the application of the TPB from different perspectives. The first detailed review by Godin and Kok (1996) [13] examined the application of the TPB in the health field, which verified that the TPB performed well in predicting and interpreting health-related behaviors, and that perceived behavioral control significantly improved the predictive ability of the model. In the same vein, Conner and Armitage (1998) [11] reviewed the relevant evidence on additional variables and summarized the expansion path in order to support the extension of the TPB in various ways for different fields. Furthermore, a meta-analysis of 185 empirical articles by Armitage and Conner (2001) [14] revealed that the TPB could account for up to 39% and 27% of the variance in intention and behavior, respectively. A recent study by Scalco et al. (2017) [15] reviewed studies applying the TPB to predict organic food consumption. Overall, these studies have examined the application and efficiency of the TPB in several domains via meta-analysis or systematic review. However, the application progress and current status of the TPB in environmental science, which are important for future environmental behavior research, are still unclear.

This study aimed to comprehensively understand the application of the TPB in the field of environmental science over the past few decades. A bibliometric analysis method, which has been widely used to map knowledge domains in various fields [16,17,18,19], was employed to aid the authors’ retrospective analysis. The primary aims of this paper are: (1) to summarize and quantify the history of environmental science research using the TPB; (2) to reveal the overall application status of the TPB in the field of environmental science from the perspective of cross-disciplines, source journals, countries and regions, article citations, and keyword co-occurrence; (3) to propose a fresh, integrated, and comprehensive knowledge framework for the application of the TPB in environmental science research, including the current topic distribution, theory integration, extended factors, major methods, specific groups, and control variables; and (4) to provide an in-depth and critical analysis of the state of the art, as well as identify the gaps, challenges, and potential directions for future research. It is hoped that this study may present more comprehensive information to aid subsequent researchers in quickly understanding the current knowledge body of the TPB and inspire their research into future TPB-based applications in environmental science and other related domains.

## 2. Research Methodology

### 2.1. Bibliometric Analysis

Bibliometric analysis, a popular method for the quantitative analysis of documents published in specific areas [20], was adopted in this study to quantify and visualize the application of the TPB in environmental science research. It employs multiple mathematical and statistical techniques to analyze the literature characteristics of a particular topic; assess the performance of authors, institutions, countries/regions, and journals; discover research hot spots; and uncover future research trends [21]. Such techniques comprise co-authorship analysis, co-occurrence analysis, citation and co-citation analysis, and the mapping of the knowledge domain [22]. It should be noted that although bibliometric analysis can extract and visualize key information from a large number of documents and provide an objective reference for retrospective analysis, it cannot replace systematic manual reviews [19,23]. Generally, comprehensive discussions based on the results of bibliometric analysis should be conducted to provide critical reviews. Regarding research tools, VOSviewer was employed to perform the bibliometric analysis in this article. VOSviewer, developed by Leiden University in the Netherlands, is a professional tool used for structuring and visualizing bibliometric networks, and it also provides text mining functions that can be employed to build and visualize the co-occurrence networks of significant labels extracted from numerous scientific documents [24]. Compared with other software programs, such as CiteSpace, BibExcel, and SciMAT, VOSviewer has unique advantages in mapping knowledge domain displays. VOSviewer’s layer label structure can clearly display dense network node interactions, making it suitable for analyzing complex networks and large-scale data generated by a large number of citations and keyword co-occurrences [25].

### 2.2. Content Analysis

Content analysis is a research method that is used to perform objective, systematic, and quantitative analysis of the literature. The essence of content analysis is the analysis of information contained in the literature and its changes, i.e., inferring the exact meaning from the meaningful words and sentences represented [26]. Content analysis is a process of layered reasoning, and researchers commonly employ the core words and sentences of literature as the basis of analysis. The content analysis method takes the qualitative hypothesis as the starting point and adopts a quantitative statistical method to process the research object [27]. The final results are qualitative conclusions from the statistical data. The research object of content analysis is the information characteristics of document content, which includes not only explicit information content, but also potential or implicit information content [28]. In this study, content analysis was used to identify and analyze the important content of keyword co-occurrence results to reveal the current application status of the TPB in many aspects of environmental science.

**Figure 2:**
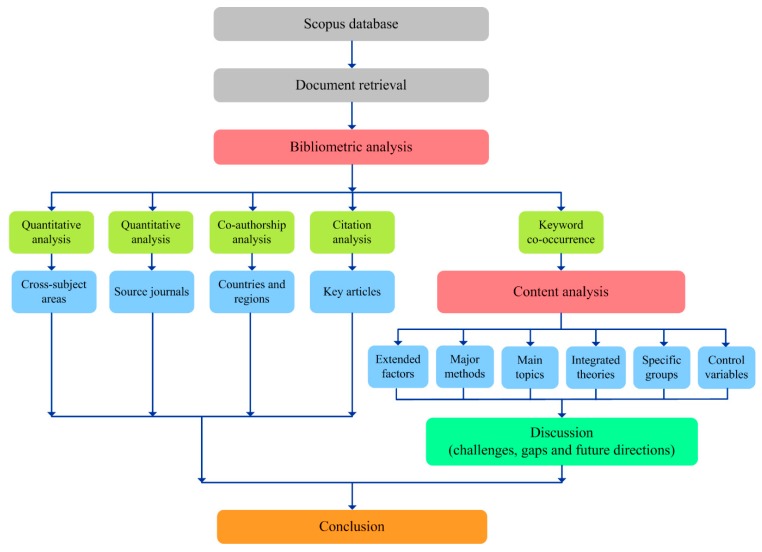
Research framework of the current study.

Based on the aforementioned information, the bibliometric analysis method and content analysis method were the primary methods used to achieve the goals of this study. Previous bibliometric studies have generally followed a specific analytical framework [19,29,30,31]. Specifically, this study was carried out according to the following steps: (1) quantitative analysis was conducted to present the cross-subject areas and source journals of 531 articles; (2) co-authorship analysis was conducted to identify the most productive countries and regions; (3) citation analysis was performed to identify the key articles; and (4) keyword co-occurrence and further content analysis were performed to uncover the main topic, major methods, extended factors, integrated theories, specific groups, and control variables. Based on the above analysis, a comprehensive discussion will be presented. The detailed research framework is shown in Figure 2.

## 3. Information Retrieval

### 3.1. Document Identification and Extraction

It is commonly acknowledged that Scopus and the Web of Science are two of the most prestigious and influential databases in the world. These two databases cover both published documents and citation databases in all fields of science, and have become valuable tools for bibliometric analysis [32]. A comparison by Vieira and Gomes (2009) [33] revealed that Scopus provides 20% more coverage than the Web of Science. Therefore, the literature in this study was retrieved from Scopus.

Specifically, we began by setting the query string to “TITLE-ABS-KEY (“theory of planned behavior” OR “theory of planned behavior”)”, and the search time was 16 March 2019 (article publication time was unlimited). Second, the source type was limited to journal articles because they have undergone rigorous peer review and are more reliable than conference papers and other types of documents. Third, the subject area was limited to "Environmental Science", as identified and classified by Scopus. It must be explained that all the documents in Scopus are divided into 27 major subject areas according to their research content, and one of these subjects is environmental science. Furthermore, the subject of environmental science in Scopus covers 13 subfields, including ecological modeling; ecology; environmental chemistry; environmental engineering; environmental science (miscellaneous); general environmental science; global and planetary change; health, toxicology and mutagenesis; management, monitoring, policy, and law; nature and landscape conservation; pollution, waste management and disposal; and water science and technology. Therefore, the search scope of this study basically covers the field of environmental science. Finally, a manual examination was performed to eliminate irrelevant literature, and several papers were excluded due to a lack of correlation. As a result, a total of 531 relevant article records from 1995 to 2019 were exported for further statistical analysis. Each record contained authors, country/region, article title, year, source title, citation count, abstract, author keywords, index keywords, and references.

### 3.2. Preliminary Literature Statistics

**Figure 3:**
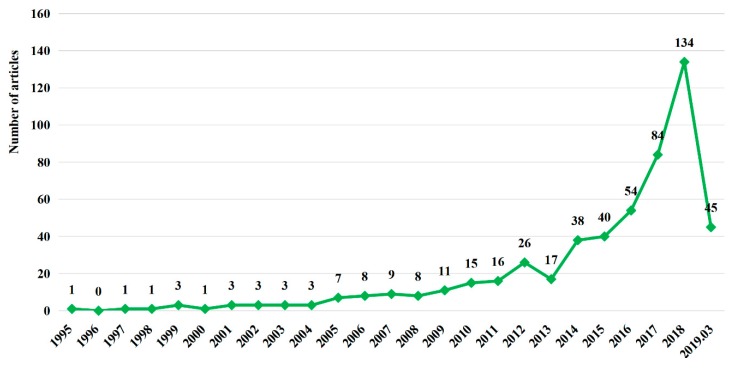
Literature statistics for the theory of planned behavior (TPB) as applied to environmental science.

Figure 3 displays the annual literature statistics for the TPB as applied to environmental science. It can be seen that the first article in the field of environmental science using the TPB appeared in 1995, and the number has significantly increased since 2008, reaching the highest number, 134 articles, in 2018. From 2008 to 2018, the average annual growth rate of publications reached 62.19%. In 2019, 45 articles were published in less than three months, which means that there may be more than 180 articles published this year. As the world pays increasing attention to human environmental behavior, the application of the TPB in future environmental science research will continue to increase and involve more topics. Therefore, it is necessary to look back on the research on the application of the TPB.

## 4. Bibliometric Analysis

### 4.1. Quantitative Analysis: Cross-Subject Areas and Primary Source Journals

**Figure 4:**
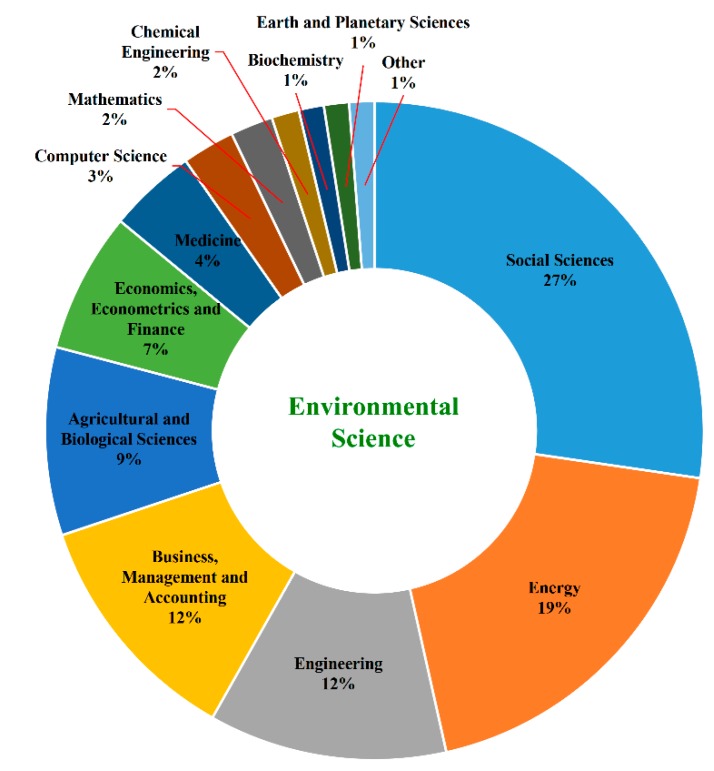
Interdisciplinary analysis.

According to Scopus’s classification system for subjects, a document may belong to more than one subject area. Indeed, 531 articles in the field of environmental science belonged to 16 other subjects, including social sciences; energy; engineering; business; management and accounting; agricultural and biological sciences; economics, econometrics and finance; medicine; computer science; and other subjects. The detailed distribution is shown in Figure 4.

Among the 531 articles applying the TPB, 58% of the papers are interdisciplinary studies between environmental sciences and social sciences, energy and engineering, and 28% of the papers are interdisciplinary studies involving environmental sciences and business, management, agriculture, and economics. To some extent, these disciplines represent the three pillars of sustainable development (i.e., economy, society, and environment), which implies that the TPB has played some role in promoting cross-subject area research among the three pillars. In the future, this type of research, which is intersected by environmental psychology and other disciplines, will continue to expand.

The 531 articles searched in this paper were published in 148 different journals. Table 1 lists the top 20 journals with respect to number of publications, which accounted for approximately 62% of the 148 journals. Three important journals in the domain of environmental science and sustainable development—the *Journal of Cleaner Production, Sustainability*, and *Resources Conservation and Recycling*—were ranked in the top three regarding the number of articles published. Among them, the *Journal of Cleaner Production* published 56 articles (10.55%) and was ranked first in terms of publication number; in additional, *Sustainability* was ranked second in publication number. Compared with the *Journal of Cleaner Production* and *Sustainability, Resources Conservation and Recycling* published a smaller number of articles per year, ranking third in the number of publications applying the TPB to environmental science. In addition, the publication numbers of the *Journal of Environmental Management, Land Use Policy*, and *Environment and Behavior* are comparable, ranking fourth, fifth, and sixth, respectively. These six journals accounted for 36% of the total number of publications, which suggests that they are the most influential journals applying the TPB to environmental science over the past decades. As seen from the list of the top 20 journals, in addition to environmental journals, journals on topics such as land, energy, transportation, and health also include relevant studies focusing on environmental behavior.

**Figure 5:**
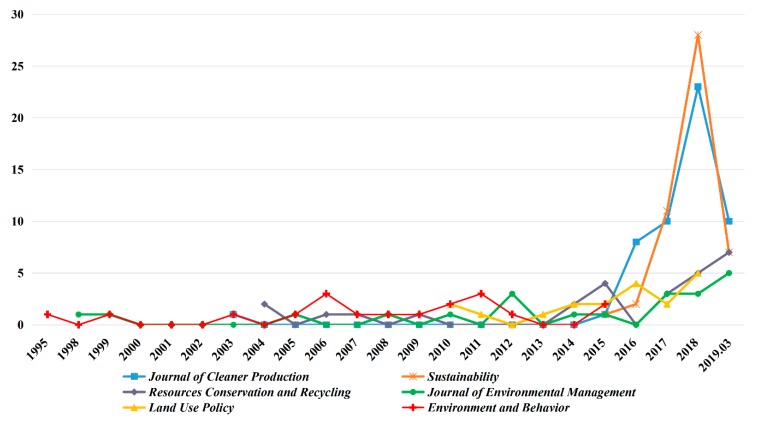
Publication statistics on the application of the TPB to environmental science.

To clearly reveal the publication status of the TPB in environmental science research in each journal, this study analyzed the publication history of the top six source journals according to the statistics of 531 articles, as displayed in Figure 5. The first article on the application of the TPB to environmental science was published in Environment and Behavior in 1995, but this journal did not publish relevant articles from 2016 to 2018. In contrast, there have been an increasing number of relevant articles published in Sustainability and the *Journal of Cleaner Production*, especially in the past four years. In addition, the number of related studies published in the *Journal of Environmental Management* and *Resources Conservation and Recycling* has gradually increased since 2016. Although *Land Use Policy* is not a journal in the field of environmental science, since 2014, it has published several studies on the application of the TPB to environmental science every year, including an investigation of farmers’ adoption of green fertilizer [34,35] and studies on agri-environmental schemes [36,37], which are all related to the topics of farmers and land. Notably, the publishing trends of these journals shown in Figure 5 are closely related to their annual total number of publications. For example, the average annual publication number of *Environment and Behavior* is between 40–50 articles, while that of Sustainability reached 7730 in 2018.

### 4.2. Co-Authorship Analysis: Productive Countries and Regions

**Figure 6:**
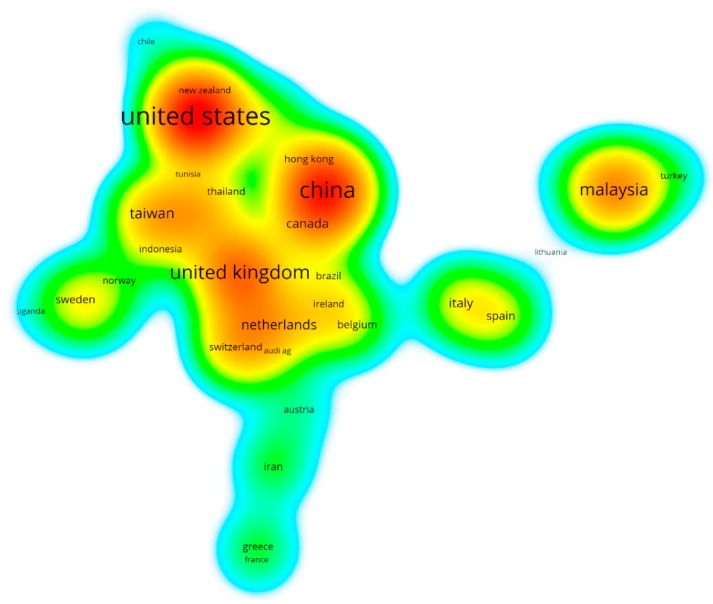
Density of the main source countries and regions.

The 531 studies on the application of the TPB to environmental science came from 69 countries and regions (the statistical method is full counting, which covers the information of all the authors in each article). Figure 6 shows the cooperative network heat map for these countries and regions (countries with a small number of productions are covered by colors). Among them, the larger the font size of the label and the redder the color of the area, the more articles that were published in that country or region. Countries/regions located in the center of the network have strong cooperation with others, while countries/regions located in a common color region have close cooperation with each other.

Table 2 details the 18 countries and regions that have published more than 10 articles and their publishing characteristics. The United States (110 articles, 20.72%), Mainland China (90, 16.95%), the United Kingdom (64, 12.05%) and Malaysia (50, 9.42%) are the four most productive countries/regions. They have made very important contributions to the application of the TPB to environmental science. Given the presence of global warming, the United States and China—as the top two economies in the world—are paying increasing attention to environmental policy practice and investing increasingly in scientific research. As China is the most populous country, the impact of human behavior on the environment has attracted increasing scholarly attention. In the United Kingdom (UK), which was one of the first countries to form environmental protection concepts and enact environmental protection laws (such as the Pigou tax), both government officials and residents place a high degree of importance on the environment [38]. Therefore, related research in environmental science has always been the focus of UK scholars. As a striking emerging economy in Asia, Malaysia has been vigorously developing tourism in recent years, given its unique climate and geographical location. Local governments (such as Kuala Lumpur) attach great importance to human settlement and sustainable consumption practices; thus, Malaysian scholars have increasingly published in academic journals on environmental behavior and sustainability topics [30,39].

In terms of citations (including self-citations and other citations), the United Kingdom (1790 citations), the United States (1779 citations), Germany (871 citations) and Mainland China (714 citations) have the highest total citations, indicating that these countries/regions have a relatively strong influence on the application of the TPB to environmental science research. Germany (38 citations/articles), the United Kingdom (28 citations/articles), and Canada (20 citations/articles) have higher than average citation numbers. With regard to cooperation, the United Kingdom (20 links), the United States (17 links), and Mainland China (16 links) have the most links, indicating that the cooperation network among the authors of these three countries/regions and their neighboring countries shown in Figure 6 is more intensive than elsewhere. It should be noted that the above analysis is based on the application of the TPB to environmental science.

### 4.3. Citation Analysis: Key Articles

**Figure 7:**
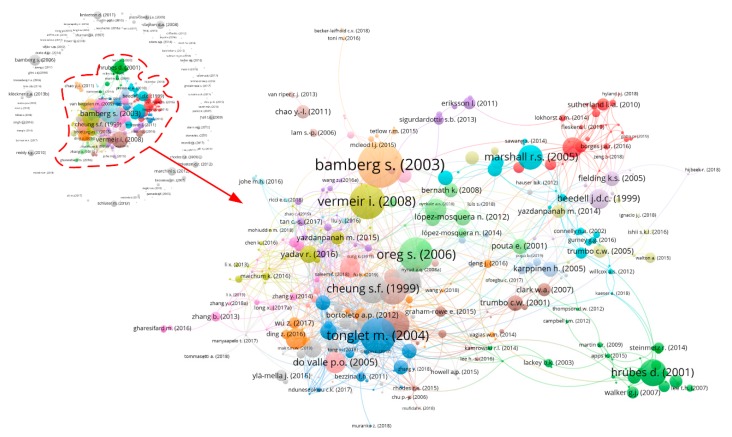
Citation network.

The upper left corner of Figure 7 is a citation network consisting of 531 articles, in which each node represents an article and the links of the connecting node represent the citation relationships. The larger the node and the denser the link, the more cited the article. These highly cited articles are considered to be the key studies in the application of the TPB to environmental science. Since different colors represent different clusters, 153 articles form a large, discrete group on the edge of the network, while the remaining 378 articles (Figure 7, lower right) form 24 focal groups comprising 1111 links. To some extent, this multigroup clustering result suggests that the research topics on environmental behavior are scattered.

Table 3 lists the top 15 articles with over 120 citations, with the bold words representing the topic of the article. The most cited article was a study published in *Environment and Behavior* by Bamberg and Schmidt in 2003, which applied the norm activation model, the TPB, and the theory of interpersonal behavior to the prediction of college students’ car use behavior and compared the predictive powers of the three theories [40]. As this study involves the application and comparison of three theories at the same time and is the earliest empirical study of car use behavior, it has been referred to and cited by many scholars. Tonglet’s study, published in *Resources Conservation and Recycling* in 2004, was ranked second in citation number. This paper investigated the decisive factors of household recycling in England based on the TPB, taking into account factors such as moral norms, outcomes, and consequences [41]. It is commendable that the research design of this article is very rigorous and reasonable. From the introduction of the research background to the discussion following the empirical analysis, the content of each chapter is very informative and worthy of being read and referred to by future generations.

The study by Vermeir and Verbeke (2007) [42], which examined sustainable consumption behavior among Belgium’s young people, was ranked third in number of citations. This study confirmed the role of consumer confidence and human values in consumer intention, and the findings provide useful insights for promoting young people’s sustainable food consumption. In addition, this article is one of the first attempts to investigate young people’s behavior using the TPB, a prelude for follow-up scholars to study other pro-environmental behaviors of young people. Based on sample surveys in 27 countries, Oreg and Katz-Gerro (2006) [43] first combined the TPB with value–belief–norm theory to predict pro-environmental behavior. This study was ranked fourth in number of citations.

Similarly, other articles predicted and explained individual behavior based on different environmental themes and focused primarily on waste recycling and management behavior, such as Cheung et al. (1999) [44], Taylor and Todd (1995) [45], Tonglet et al. (2004a) [46], Chan (1998) [47], and Begum et al. (2009) [48]. This phenomenon indicates that different types of waste recycling behavior have been the focus of scholars in the past few decades.

Note that of the 15 highly cited articles, only Klöckner (2013) [4] was published after 2010. This recent study, based on testing 56 different data sets, proposed an integrated model that includes the TPB, normative activation models, and value–belief–norm theory as a framework for identifying predictors of different types of environmental behavior. This study received many citations in a short period of time, indicating that it is being recognized by an increasing number of scholars. In addition, 15 articles were considered key research contributions in the application of the TPB to the field of environmental science, while six of these articles were published in *Environment and Behavior*. To a certain extent, this issue indicates that the papers published in *Environment and Behavior* are of high quality and have a significant influence.

### 4.4. Keyword Co-Occurrence Analysis: Main Topics, Major Methods, Extended Factors, Integrated Theories, Specific Groups, and Control Variables

**Figure 8:**
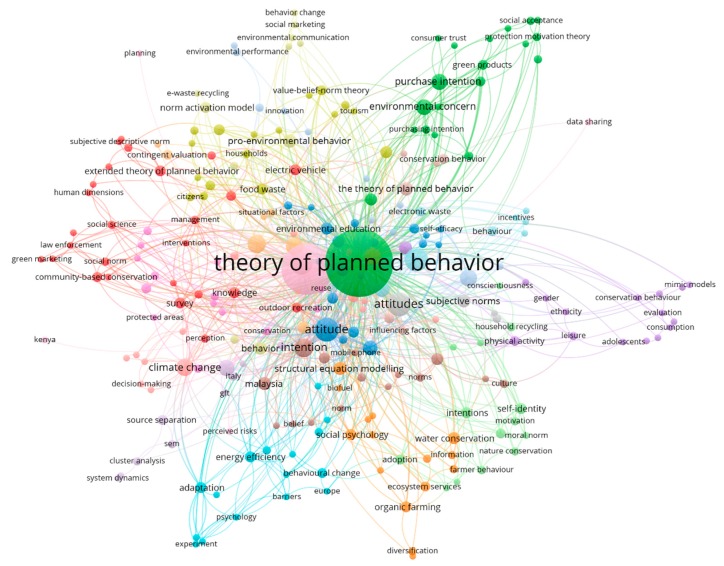
Author keyword co-occurrence network.

Keywords are descriptive words that briefly reflect the theme, method, and content of an article. The 531 articles retrieved in this study contain 1537 keywords. A total of 252 keywords appearing more than twice were selected for a co-occurrence analysis consisting of a keyword co-occurrence network with 252 nodes and 1071 links. These keywords are divided into 19 clusters of different sizes, as shown in Figure 8. The sizes of the nodes and tags are proportional to the frequency of keyword occurrence. Not surprisingly, the most frequently occurring words are “theory of planned behavior” (190 times) and “theory of planned behaviour” (110 times), as they are the search terms used in this study; “attitude” and its plural ranked second (48 times), indicating that attitude is one of the key predictors in current research on TPB application in environmental science; “recycling” (23 times) ranked third, indicating that recycling behavior is the main theme of TPB research in the field of environmental science; and the fourth is “structural equation modeling” (21 times), which is an empirical method that is widely used in TPB research and considers the mainstream questionnaire research tool of the past decade. It should be pointed out that the above keywords illustrate part of the current status of the application of the TPB in environmental science according to four aspects: theory, construct, research topic, and method. In fact, the 252 keywords appearing more than twice also contain a significant amount of other research information.

**Figure 9:**
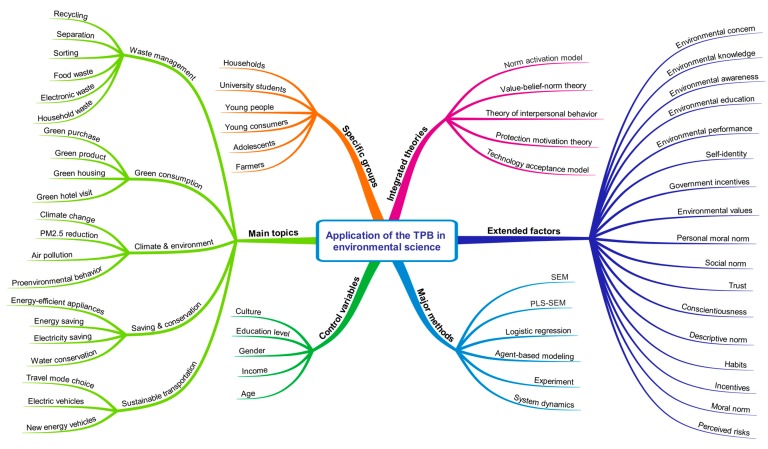
Application framework of the TPB in environmental science: 1995–2019.

In addition, the 19 clusters generated by VOSviewer are shown in Figure 8 in different colors. However, these generated clusters are so scattered and unclear that they are difficult to further analyze. Considering that empirical research on the application of the TPB to environmental science includes different topics, methods, theoretical integration, extended factors, control variables, and specific groups, they and many related terms have also appeared in the keywords of articles. The content analysis method takes the qualitative problem hypothesis as the starting point and adopts a quantitative statistical method to process the research object, which includes not only explicit information content but also potential or implicit information content [28]. Therefore, using the content analysis method, this study attempted to extract and classify 252 keywords from the aspects of topic, method, theory, factor, and group to provide a comprehensive application framework for future relevant research. The results are shown in Figure 9.

#### 4.4.1. Main Topic Distributions

Based on the explicit and implicit information content presented by 252 keywords, this paper extracts and divides the research topic into five categories: waste management, green consumption, climate and environment, saving and conservation, and sustainable transportation. With regard to waste management, recycling, separation, and sorting are the main treatment measures; food waste, electronic waste, and household waste, as well as construction and demolition waste, are the primary types of waste. For example, Mak et al. (2018) [54] examined food waste recycling practices in the commercial and industrial sectors by extending the TPB. Recent studies by Thi et al. (2019) [55] and Wang et al. (2019) [56] investigated the determinants of residents’ willingness to recycle e-waste. Zhang et al. (2019) [57] and Fan et al. [58] performed an empirical analysis of the intention and behavior of household waste classification in China. The results revealed that individual norms and environmental motivation were considered to be the critical influencing factors. Similarly, based on the extension of the TPB, Mak et al. (2019) [59] explored the critical factors for the recycling behavior of construction and demolition waste stakeholders in Hong Kong.

In terms of green consumption, the research topics in existing research applying the TPB include green purchases, green products, green housing, and green hotel visits. Typical studies are as follows: Chen and Deng (2016) [60] surveyed people’s green purchasing intention from the perspective of product knowledge. Maichum et al. (2016) [61] investigated Thai consumers’ willingness to purchase green products using environmental knowledge and environmental concerns as antecedents of the TPB model. Yadav and Pathak (2017) [62] identified the key factors of consumers’ green purchasing behavior in developing countries by expanding the TPB. Zhang et al. (2018a, 2018b) [63,64] studied the purchase intention of young Chinese consumers regarding green housing, pointing out that the attitude of governments and consumers is the most important factor. Verma and Chandra (2018) [65] predicted Indian consumers’ intention to visit green hotels by extending the TPB, demonstrating the importance of moral reflection and responsibility.

With regard to climate and environment, climate change, pro-environmental behavior, PM2.5 reduction and air pollution are the main keywords. Specifically, Lin (2013) [66] investigated the pro-environmental behavior of Kaohsiung residents using the TPB, and found that attitude toward global warming can affect citizens’ pro-environmental behavior intention. Arunrat et al. (2017) [67] examined the intention and decision of Thai farmers to adapt to climate change using empirical research, which highlighted that behavior control is the key influencing factor. Chuang et al. (2018) [68] integrated the TPB and Unified Theory of Acceptance and Use of Technology to predict Taiwanese tourists’ pro-environment behavior intention. Zahedi et al. (2019) [69] explored the willingness of Catalonians to reduce air pollution and greenhouse gases, and discovered that most respondents were willing to pay for it. Ru et al. (2019) and Shi et al. (2017) [70,71] analyzed the PM2.5 reduction behavior of Chinese families and adolescents by extending the TPB, and found that attitudes and perceived behavioral control had a critical impact on PM2.5 reduction behavior.

With respect to saving and conservation, keywords in this field include energy-efficient appliances, energy saving, electricity saving, and water conservation. Over the past decade, the environmental problems caused by global energy and resource consumption have prompted governments and scholars to pay increasing attention to individual energy-saving, power-saving, and water-saving behaviors. For instance, Tan et al. (2017) and Li et al. (2019) [72,73] respectively extended the TPB from the level of ethics and environmental concerns and predicted the purchasing intention of Malaysian and Chinese consumers for energy-efficient appliances. Gao et al. (2017) and Ru et al. (2018) [74,75] explored the individual’s willingness to save energy through the extended TPB and recognized the importance of normative factors and perceived behavior control. Yazdanpanah et al. (2016) [76] took into account morality and self-identification to investigate the willingness of young Iranians to save water. Taking office workers as an example, Zhang et al. (2014) [77] assessed the decisive factors of employees’ electricity-saving behavior.

In relation to sustainable transportation, travel mode choice, electric vehicles, and new energy vehicles are major research topics. Sustainable transportation plays a critical role in promoting the economic growth of cities and the living environment of residents [19]. Many countries, including China, are increasingly aware of the importance of green travel. Wall et al. (2007) [78] compared the norm activation model with the TPB to reveal drivers’ willingness to reduce or maintain commuting through vehicles. Birna et al. (2013) [79] carried out an empirical analysis of teenagers’ willingness to commute with cars or bicycles and asserted that car use experience and cycling experience play an important role. Zhang et al. (2018) and Huang and Ge (2019) [80,81] analyzed the buying intention of Chinese consumers from the perspective of government incentives. By extending the TPB, Wang et al. (2017) [82] examined the factors influencing Chinese residents’ new energy vehicle purchase behavior.

#### 4.4.2. Integrated Theories, Extended Factors, and Control Variables

Widespread in social psychology, the norm activation model (NAM) [83], value-belief-norm theory (VBN) [84], theory of interpersonal behavior (TIB) [85], protection motivation theory (PMT), [86] and the technology acceptance model (TAM) [87] are usually integrated with the TPB to conduct empirical research in the environmental science field. For example, Liu et al. (2017) [88] combined the NAM and TPB to propose a comprehensive model to investigate the sustainable transport behavior of 600 Chinese drivers. Zhang et al. (2017) [89] integrated the NAM and TPB to survey Chinese citizens’ environmental complaint behavior. Based on the TPB and VBN, a comprehensive model to predict transnational pro-environmental behavior was proposed and tested by Oreg and Katz-Gerro (2006) [43]. Sung and Cooper (2019) [90] integrated the TIB and TPB to examine the critical factors influencing UK manufacturer upcycling behavior. Wang et al. (2019) [91] discussed the determinants of farmers’ environmental behavior and willingness in China’s water conservation areas by integrating the TPB and PMT. Zheng et al. (2019) [92] incorporated perceived usefulness and perceived usability of the TAM into the TPB model and explored the rental behavior and intention of the younger generation in Jinan, China.

In addition to considering integrating the TPB with other theories, existing empirical studies on environmental behavior generally extend two to five other variables into TPB models to explain and predict specific behaviors more comprehensively. The extension factors shown on the right side of Figure 9 are some of the commonly used latent variables, especially environmental concern, environmental knowledge, environmental awareness, and environmental education. For instance, Choi and Johnson (2019) and Li et al. (2019) [73,93] added environmental concerns and environmental knowledge to TPB models to understand consumers’ purchasing intention for energy-efficient appliances. Taking into account environmental awareness, environmental concerns, and environmental knowledge, Fu et al. (2019) [94] explored factors influencing public willingness to improve air quality. Mohiuddin et al. (2018) [95] determined the impacts of environmental awareness on students’ willingness to buy green vehicles with a TPB framework. Leelapattana et al. (2019) [96] used environmental education as an antecedent variable of the TPB to reveal the impact of environmental education on consumers’ intention when choosing a farm vacation. In addition, other extension factors are often used in related studies, such as environmental performance [97,98,99], self-identity [76,100], government incentives [64,92], and environmental values [101,102].

Additionally, some control variables such as culture, education level, gender, income, and age were identified in the questionnaire, and are normally used for a comparative analysis of the moderating effects or multigroup results. For example, Geiger and Schrader (2019) [103] examined the cultural differences between Tehran and Berlin in the context of collaborative fashion consumption through the TPB. Mandravickait and Bernatonien (2016) [104] conducted a cross-cultural exploration of the decisive factors of green purchasing behavior of consumers in European Union (EU) countries through the TPB. Huang and Ge (2019) [81], exploring Beijing consumers’ willingness to buy electric cars, analyzed and compared samples of individuals of different gender, age, income, and education levels in a multigroup manner and found some significant differences. In the same vein, Hu et al. (2019) [105] used gender, age, and education level as control variables to analyze the intention of tourists to participate in the zero-waste plan in tourist areas and its influencing factors.

#### 4.4.3. Specific Groups and Major Methods

In addition to the general respondent groups such as consumers and the public, there are also some specific groups in articles on the TPB as applied to environmental science, including households, university students, young people, young consumers, adolescents, and farmers. These specific groups have attracted much attention in the past five years. For example, Zhang et al. (2019) [106] evaluated the willingness of households in Beijing to pay for green roofs to mitigate the urban heat island effect. Fan et al. (2019) [58] carried out a comparative analysis on the intention and behavior of Chinese and Singaporean households to classify solid waste. Sun et al. (2015) [107] conducted an empirical study on the walking behavior of college students in Hong Kong using the TPB and structural equation modeling (SEM). Ru et al. (2019) [70] explored the behavioral intention of young people in China to reduce PM2.5 by extending the TPB model. Yadav and Swaroop (2016) [108] investigated the green product purchase intention of young consumers in developing countries. Verma and Chandra (2018) [65] predicted the willingness of young Indian consumers to visit hotels.

With regard to research methods, covariance-based SEM and partial least squares structural equation modeling (PLS-SEM) are the two most commonly used methods in TPB applications. The former is mainly applicable to the analysis of the causal relationship of potential structures to verify specific theories, while the latter is more suitable for the development and prediction of theories. Detailed applicable situations, advantages, and disadvantages of SEM and PLS-SEM were elaborated by Hair et al. (2014 and 2011) [109,110]. In addition, logistic regression, agent-based modeling, experimentation, and system dynamics also appeared in the application of the TPB in environmental science research. For instance, Arunrat et al. (2017) [67] adopted a logistic regression model to examine farmers’ intention to adapt to climate change. Mashhadi (2018) [111] proposed an agent-based modeling framework based on TPB to simulate consumers’ product usage decisions. Tong et al. (2018) [112] surveyed the determinants of household recycling behavior by combining a social experiment and agent-based modeling. Based on the TPB, Ding et al. (2016) [99] established a system dynamics model of construction waste reduction management in the construction stage of the project and simulated the environmental benefits of reduction solutions using project data for Shenzhen, China.

## 5. Discussion: Current Challenges, Gaps, and Future Directions

Global environmental issues are directly related to human health, economic prosperity, and social well-being. The latest Global Environmental Outlook released by the United Nations reports that human behavior has a variety of impacts on biodiversity, the atmosphere, oceans, water, and land, resulting in serious and even irreversible environmental degradation, which in turn threatens human health [113]. With the global emphasis on human environmental behavior issues, as a common theory for predicting and interpreting individual behavior and its influencing factors, the TPB has attracted widespread attention from researchers in various fields. Nevertheless, some issues need to be reflected in the application of the TPB in environmental science. Based on the current application status revealed in this study and systematic content analysis, we will conduct an in-depth and critical analysis of the state of the art and identify primary challenges, potential gaps, and directions for future research.

As presented in the application framework in Figure 9, the TPB has been employed in a wide range of behavioral research on environmental science topics and covers a wide variety of groups. However, the current research is mainly focused on the perspective of ordinary residents and consumers. Due to the difficulty of data acquisition, few studies have considered the investigation of environmentally relevant behaviors of decision-makers and designers, such as enterprise leaders, government officials, and product designers. In fact, as one of the United Nation (UN)’s sustainable development goals, sustainable production and consumption are largely dependent on sustainable decision making and green design [114,115,116]. In other words, the intention and behaviors of decision-makers and designers toward cleaner production, sustainable operation, and policy implementation are more important and meaningful than those of consumers. Therefore, it is necessary to predict and reveal the intention and behavior of business leaders, designers, and government officials using the TPB and related theoretical extensions. In addition, the PLS-SEM method, which requires fewer samples than covariance-based SEM [109,110], may mitigate the challenge of data collection.

A major contribution of TPB application research is the provision of practical implications for the guidance of specific behaviors; however, the empirical results of many TPB-based environmental behavior studies are inconsistent, despite having the same topic and background. For example, Ru et al. (2019) and Shi et al. (2017b) [70,117] reported that subjective norms have positive impacts on Chinese residents’ behavioral intention toward reducing PM_2.5_. However, in another study by Shi et al. (2017a) [71], the authors pointed out that the effects of subjective norms on the PM_2.5_ reduction intention of Chinese households are not obvious. Similarly, regarding residents’ intention toward new energy vehicle purchases in China, Wang and Dong (2016) [118] claimed that subjective norms have significant positive impacts on intention, while Huang and Ge (2019) [81] revealed the opposite findings. With the increasing number of empirical studies on pro-environmental behavior by employing the TPB and related theories, such inconsistent conclusions may appear frequently in the future. Thus, these theories cannot reliably predict individual behavior or guide practice, and may mislead policymakers. There are two ways to address this challenge. Scholars need to explain the research differences as much as possible and explore the root causes of the differences. However, since self-reported questionnaires may lead to biased answers [119], with the progress of information technology and medical equipment, more accurate physiological experiments and other interdisciplinary methods should be adopted and integrated with existing methodology.

In terms of current research groups, while existing studies have primarily surveyed only one or two groups, few studies have considered making comparisons of multiple groups, such as multi-stakeholder groups and different groups in developed and developing countries. Such comparative analyses of the behavioral psychological factors of different stakeholders and economic and social backgrounds would seem to be more helpful for policy making. For instance, recycling and reduction behaviors for construction and demolition waste involve many stakeholders, such as contractors, developers, suppliers, governments, designers, waste disposal stations, and the public, and economic development and technical level vary from country to country. Nevertheless, although the comparative results based on the above groups are unclear, they are important for facilitating more comprehensive decisions and providing implications for developing countries. Therefore, multigroup comparison research from the perspective of multi-stakeholder and multi-country perspectives based on the TPB is needed in environmental science.

Regarding the TPB and its extensions, to date, numerous studies have tried to improve the interpretation ability of the theory by extending variables or integrating other theories into the TPB model. However, the research on influencing factors predicted by extending the TPB has shown limitations to varying degrees. It must be noted that the TPB is a sociopsychological theory for explaining individual willingness and behavior, and it can well predict the psychological driving factors in the execution of a particular behavior. Whether the existing predictors can fully represent the influencing factors of a particular behavior needs further consideration. Since many studies examined the influencing factors of a particular behavior only by the TPB or its extensions [94,120,121], some external factors and demographic factors may be overlooked. In the future, environmental science research applying the TPB needs to utilize additional theories and variables in interdisciplinary fields and consider more related external factors and demographic factors to present better predictions and implications.

In March 2019, negotiations and consultations at the Fourth United Nations Environment Congress focused on such major issues as curbing food wastage and resolving the crisis of marine plastic pollution [122]. With respect to research topics in environmental science, while a considerable number of studies have focused on recycling behavior by extending the TPB, far too little attention has been paid to specific behaviors related to plastic use and food wastage. There is increasing evidence that the use and disposal of plastics have caused serious pollution in terrestrial and marine ecosystems [123]. Since up to eight million tons of plastic waste flow into the ocean every year, a solution to the marine plastic pollution crisis is urgently needed [113]. Moreover, approximately 33% of the world’s edible food is lost or wasted, approximately 56% of which occurs in developed countries [113]. Global food waste is not only related to the reduction in supply but also has a direct impact on the environment, such as greenhouse gas emissions, surface and underground water consumption, and land occupation [124]. Given the above findings, plastic use and food wastage should be afforded sufficient attention in the future, with a focus on usage behavior avoidance, usage behavior reduction, and plastic recycling behavior, as well as food saving and waste reduction behavior.

## 6. Conclusions

This study comprehensively analyzed 531 papers on the application of the TPB to environmental science in the past 25 years using bibliometric and content analysis. Specifically, the interdisciplinary situations and source journals were quantitatively analyzed, and the most productive countries and regions were revealed through co-authorship analysis, as well as their network distribution. The citation networks and 15 key articles were identified via citation analysis. More importantly, based on keyword co-occurrence and manual classification, this study elaborated the main research topics, extended factors, integrated theories, major methods, specific groups, and control variables of the TPB as applied to the field of environmental science. A critical analysis of current challenges, gaps, and future directions was ultimately discussed. Overall, this study revealed some interesting findings.

### 6.1. Main Research Findings

First, the quantitative analysis results by the subject area and the co-occurrence analysis results of the keywords all revealed rich research themes and interdisciplinary interactions. This finding indicates the universal applicability of the TPB in predicting various individual behaviors and it also confirms that the intersection of environmental science subjects and other disciplines will become a new trend in the development of environmental behavior research, especially in the fields of social science, energy, and engineering, as well as business, management, agriculture, and economics. In addition, from 2008 to 2018, the average annual growth rate of 62.19% in the number of articles indicates that the TPB will be more widely used in environmental science and related fields in the future.

Second, the *Journal of Cleaner Production, Sustainability, Resources Conservation and Recycling, Journal of Environmental Management, Land Use Policy, Journal of Environmental Management, Land Use Policy*, and *Environment and Behavior* (ranked by number of publications) are the main journals for publishing environmental science research using the TPB. Notably, 105 of the 531 articles (19.78%) were published in the *Journal of Cleaner Production and Sustainability*, and the number of publications in these two journals has increased rapidly over time. In addition, the number of publications on environmental science research using the TPB in *Resources Conservation and Recycling, Journal of Environmental Management*, and *Land Use Policy* has increased in the past five years.

Third, the United States, Mainland China, the United Kingdom, and Malaysia are the four most productive countries/regions that have made very important contributions to environmental science research using the TPB. The possible reasons for this include national culture, habits, economics, and policies. In contrast, studies from the United Kingdom, the United States, Germany, and Mainland China have been cited the most frequently and are more influential. The research networks in the United Kingdom, the United States, and Mainland China are more interconnected, and scholars in these countries/regions are interested in cooperating with scholars from other countries.

Fourth, ranked according to the number of citations, Bamberg and Schmidt (2003) [40], Tonglet et al. (2004b) [41], Vermeir and Verbeke (2007) [42], Oreg and Katz-Gerro (2006) [43], Cheung et al. (1999) [44], Taylor and Todd (1995) [45], Klöckner (2013) [4], Tonglet et al. (2004a) [46], Hrubes et al. (2001) [49], Chan (1998) [47], Marshall et al. (2010) [50], Bamberg (2006) [51], Spash et al. (2006) [52], Valle et al. (2005) [53], and Begum et al. (2009) [48] are the 15 most cited articles. They play critical roles in guiding the application and development of the TPB in the field of environmental science. Surprisingly, six of these 15 key articles were published in *Environment and Behavior*, which has published a total of 18 articles on the application of the TPB in environmental science. To some extent, this finding suggests that the articles published in *Environment and Behavior* are of high quality and enjoy widespread recognition in the research field of environmental behavior.

Fifth, regarding the application of the TPB to the field of environmental science, the topics involved mainly include waste management, green consumption, climate and the environment, saving and conservation, and sustainable transportation. The theories used for integration mainly include the NAM, VBN, TIB, PMT, and TAM. The main factors used for model extension include environmental concern, environmental knowledge, environmental awareness, environmental education, environmental performance, self-identity, government incentives, and environmental values. The control variables used for multi-group comparison primarily include culture, education level, gender, income, and age. In addition to the general respondents such as consumers and the public, the objects studied include households, university students, young people, young consumers, adolescents, farmers, and other groups. In addition to SEM and PLS-SEM, the methods employed include logistic regression, agent-based modeling, experimentation, and system dynamics.

### 6.2. Contributions

Through an integrated bibliometric analysis, the results of this study provide, for the first time, a comprehensive representation of the application of the TPB to environmental science. The findings complement the TPB and environmental psychology and lay several foundations for future research. Specifically, the discovery of interdisciplinarity provides inspiration and motivation for scholars in different fields to conduct interdisciplinary individual behavior research. The quantitative analysis of source journals guides researchers in selecting target journals, examining the literature, and submitting manuscripts. The identification of 15 key articles facilitates the rapid introduction of follow-up researchers and a familiarity with environmental psychology. Moreover, the presentation of the main research topics, extended factors, integrated theories, major methods, specific groups, and control variables can provide a holistic reference framework for further study design and for writing about the application of the TPB to environment-related behavior, and it can also serve as a reference for researchers who return to the field of environmental behavior after leaving this field for a time. More importantly, a critical analysis based on systematic content analysis was conducted to reveal current challenges, potential gaps, and directions for future research, which added value to the present study.

### 6.3. Limitations

Although every effort was made to conduct a robust bibliometric analysis, this study has some limitations. First, since the data used in this study were collected from Scopus, the organization information of the articles may not be consistent, because the organization names do not have a consistent format. Therefore, this study did not analyze collaborative networks among different institutions and authors. Second, this study identified the literature on the application of the TPB in the field of environmental science through the subject classification system of Scopus, which may have resulted in a few missed articles. Third, environmental science involves many subdisciplines and subsystems, and due to the lack of integration of the authors’ academic levels, an in-depth analysis of different topics and knowledge points was not performed. In the future, more detailed manual reviews should be carried out to summarize the application of the TPB to additional fields.

## Figures and Tables

**Table 1:** Primary source journals and their characteristics.

Rank	Journal Name	No. of Articles	Percentage
1	*Journal of Cleaner Production*	56	10.55%
2	*Sustainability*	49	9.23%
3	*Resources Conservation and Recycling*	25	4.71%
4	*Journal of Environmental Management*	22	4.14%
5	*Land Use Policy*	19	3.58%
6	*Environment and Behavior*	18	3.39%
7	*Advanced Science Letters*	17	3.20%
8	*International Journal of Environmental Research and Public Health*	16	3.01%
9	*Energy Policy*	15	2.82%
10	*Ecological Economics*	13	2.45%
11	*Waste Management*	11	2.07%
12	*Forest Policy and Economics*	10	1.88%
13	*Human Dimensions of Wildlife*	9	1.69%
14	*Journal of Air Transport Management*	9	1.69%
15	*Society and Natural Resources*	9	1.69%
16	*Journal of Environmental Planning and Management*	8	1.51%
17	*Applied Environmental Education and Communication*	6	1.13%
18	*Environmental Management*	6	1.13%
19	*Science of the Total Environment*	6	1.13%
20	*Journal of Transport and Health*	5	0.94%

**Table 2:** Publication characteristics of productive countries and regions.

Country/Region	Articles	Percentage	Citations	Average Citations	Links
United States	110	20.72%	1779	16	17
Mainland China	90	16.95%	714	8	16
United Kingdom	64	12.05%	1790	28	20
Malaysia	50	9.42%	444	9	7
Taiwan	33	6.21%	346	10	4
Australia	30	5.65%	551	18	11
Netherlands	26	4.90%	521	20	14
Canada	23	4.33%	272	12	10
Germany	23	4.33%	871	38	13
Italy	21	3.95%	122	6	7
Spain	17	3.20%	260	15	3
Sweden	14	2.64%	205	15	8
Iran	12	2.26%	189	16	3
South Korea	12	2.26%	47	4	3
Belgium	11	2.07%	463	42	6
Hong Kong	10	1.88%	472	47	3
India	10	1.88%	185	19	2
Switzerland	10	1.88%	158	16	4

**Table 3:** The top 15 cited articles applying the TPB to environment science.

Document	Title	Citation	Source Publication
[40]	Incentives, morality, or habit? Predicting students’ **car use** for university routes with the models of Ajzen, Schwartz, and Triandis	434	*Environment and Behavior*
[41]	Using the theory of planned behaviour to investigate the determinants of **recycling behaviour**: a case study from Brixworth, UK	294	*Resources, Conservation and Recycling*
[42]	**Sustainable food consumption** among young adults in Belgium: Theory of planned behaviour and the role of confidence and values	283	*Ecological Economics*
[43]	**Predicting proenvironmental behavior** cross-nationally: Values, the theory of planned behavior, and value-belief-norm theory	276	*Environment and Behavior*
[44]	Reexamining the theory of planned behavior in understanding **wastepaper recycling**	205	*Environment and Behavior*
[45]	An integrated model of **waste management** behavior: A test of household recycling and composting intentions	194	*Environment and Behavior*
[4]	A comprehensive model of the psychology of **environmental behaviour**—A meta-analysis	192	*Global Environmental Change*
[46]	Determining the drivers for **householder pro-environmental behaviour**: waste minimisation compared to recycling	187	*Resources, Conservation and Recycling*
[49]	Predicting **hunting intentions and behavior**: An Application of the Theory of Planned Behavior	180	*Leisure Sciences*
[47]	Mass communication and pro-environmental behaviour: **waste recycling** in Hong Kong	169	*Journal of environmental management*
[50]	Exploring individual and institutional drivers of proactive environmentalism in the US **Wine industry**	159	*Business Strategy and the Environment*
[51]	Is a residential relocation a good opportunity to change **people’s travel behavior**? Results from a theory-driven intervention study	132	*Environment and Behavior*
[52]	Motives behind willingness to **pay for improving biodiversity in a water ecosystem**: Economics, ethics and social psychology	126	*Ecological Economics*
[53]	Combining behavioral theories to predict **recycling involvement**	126	*Environment and Behavior*
[48]	Attitude and behavioral factors in **waste management in the construction industry** of Malaysia	123	*Resources, Conservation and Recycling*

The bold words representing the topic of the article.
